# Optical Microscopy and Electron Microscopy for the Morphological Evaluation of Tendons: A Mini Review

**DOI:** 10.1111/os.12637

**Published:** 2020-02-25

**Authors:** Mingyou Xu, Jie Liu, Jiayi Sun, Xinrong Xu, Yongcheng Hu, Bin Liu

**Affiliations:** ^1^ Graduate School Tianjin Medical University Tianjin China; ^2^ Department of Orthopedic Oncology Tianjin Hospital Tianjin China; ^3^ Center for Medical Device Evaluation NMPA Beijing China; ^4^ Analytical and Testing Center South China University of Technology Guangzhou China

**Keywords:** Electron microscopy, Histology, Optical microscopy, Tendons, Ultrastructure

## Abstract

The morphological characteristics of tendons have been thoroughly evaluated via microscopy. Optical microscopy and electron microscopy are the most commonly used techniques for tendon tissue observation. According to the principles of both microscopy types, preparation and evaluation methods vary. Simple optical microscopy is commonly used in the observation of cells and extracellular matrix, and many stains, including hematoxylin–eosin, Van Gieson, Prussian blue, Alcian blue, and toluidine blue, are used for evaluating cells, collagen fiber arrangement, and noncollagenous proteins. Histological scoring systems have been used in many studies for semi‐quantification. Scanning electron microscopy (SEM) and transmission electron microscopy (TEM) are the most commonly used electron microscopy types, and special consideration is needed for the fixation and embedding protocols. Glutaraldehyde followed by osmium is most commonly used in the chemical fixation of tendon tissue, followed by epoxy resin embedment. Longitudinal sections captured in SEM images show the arrangement of collagen fibrils and the cells and lipid drops among them, while cross sections captured in TEM images show the diameter and distribution of collagen fibrils. SEM and TEM are used together for comprehensive evaluations. This mini review is focused on the preparation methodology and related evaluation indexes for the morphological evaluation of tendons.

## Introduction

The morphology and structure of tendon tissue have been thoroughly evaluated through the application of microscopy techniques. Tendons are fibrous connective tissues with hierarchical structure scales ranging from a dozen nanometers to a dozen millimeters^1^. To observe the tendon components at different scales, optical microscopy and electron microscopy are often conducted. Optical microscopes, also called light microscopes, are the most suitable devices for distinguishing cell and tissue morphology. Using visible light as an illumination source, the optical microscope is simple and inexpensive and mainly used for imaging tissue structures and cells. However, its resolution is theoretically limited to approximately one‐half the wavelength of visible light (200 nm)^2^, ^3^. In contrast to the optical microscope, the electron microscope uses electron beams as the source of illumination, and it has higher resolution and can be used to observe smaller structures than can be visualized using an optical microscope^4^. Depending on the mechanism by which the electron beam is detected, scanning electron microscopy (SEM) and transmission electron microscopy (TEM) are used to take images of a surface and section, respectively.

To observe different compositions and structures using optical microscopy, staining techniques are used, including the use of hematoxylin–eosin (HE), Van Gieson, Prussian blue, Alcian blue, and toluidine blue (TB) stains^5‐7^. In addition to these staining methods, new embedding methods for use in tendon observations have been developed, including those based on glycol methacrylate and methyl methacrylate, which offer better fidelity of collagen fiber and synovium images^8^. For electron microscopy, various fixation and staining methods are used^9^. Each method has advantages for observing molecules, cells, and tendon tissues. In addition to the preparation methods, the contents and indexes of the morphological evaluation vary greatly by the purposes of the morphological studies of tendons, and different contents are observed and evaluated according to the study design. Some studies evaluate the morphology of tendons *in vitro*, mainly focusing on the structural changes to collagen fibers or collagen fibrils with the higher magnification of an electron microscope^7^, ^10^. Similarly, in some studies on allogenic tendon treatments, resident cells are investigated^11^. On the other hand, many studies investigate tendon histology *in vivo* and evaluate both cells and vessels. In addition, the surrounding extracellular matrix and related components are commonly investigated.

The present mini literature review was aimed at briefly summarizing the preparation techniques and evaluation indexes used for the morphological analysis of tendons, providing references for further study of tendon tissue morphology.

## Optical Microscopy

### *Staining Methods for Preparation*

Before observation, sections are prepared, fixed, and embedded in resin. Staining is subsequently conducted to distinguish the different components of tendon tissue sections. The most commonly used method is hematoxylin–eosin (HE) staining, in which collagen and other extracellular matrix components are stained pink with the cell nuclei stained blue. HE stain is widely used in the first step in the verification of decellularization^11^. To investigate the damage of repeated freeze–thaw cycles to tendons, specimens are stained with Van Gieson and HE stains, which both reveal a crimpled, split, fragmented and disorganized tendon, with increases in the number of freeze–thaw cycles^7^.

**Figure 1:**
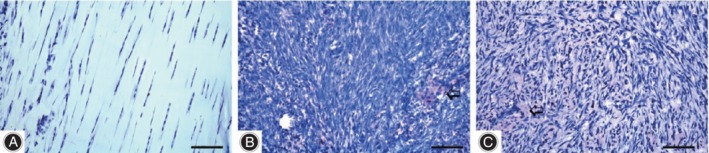
The images of TB‐stained tendon sections showed less metachromasy (arrow) in the transection region of (A) the normal group in relation to (B) the control group and (C) calendula group, with no marked difference between the transected groups. Bars = 20 μm.

To evaluate the influence of Calendula officinalis cream on Achilles tendon healing, Aro *et al*.^6^ tested specimens stained with TB and found that, in normal group, tendon matrix lacked TB staining that correlated to a low amount of proteoglycans, comparing with calendula group and control group (treated with vehicle cream after tendon transected) (Fig. 1). To study the maturation of chicken calcaneal tendons, Feo *et al*.^12^ used HE, TB, and Xylidine Ponceau stains and observed the formation of collagen fibrils in the fibrocartilage region and peripheral region during maturation. To investigate the laser‐induced denaturation effects of collagen, patellar ligaments were stained with the methylene blue (azure II) using the basic fuchsine threechrome method after laser treatment^13^. No angular crimps or smooth wavy collagen fascicles/bundles were observed in the optical microscope images. Other staining methods including Picrosirius^14^, Prussian blue^15^, Masson's trichrome^16^, and safranin orange fast green^17^ are also used.

### *Histological Analysis of Tendons in Optical Microscopy*

#### *Histological Analysis with Staining Methods*

**Figure 2:**
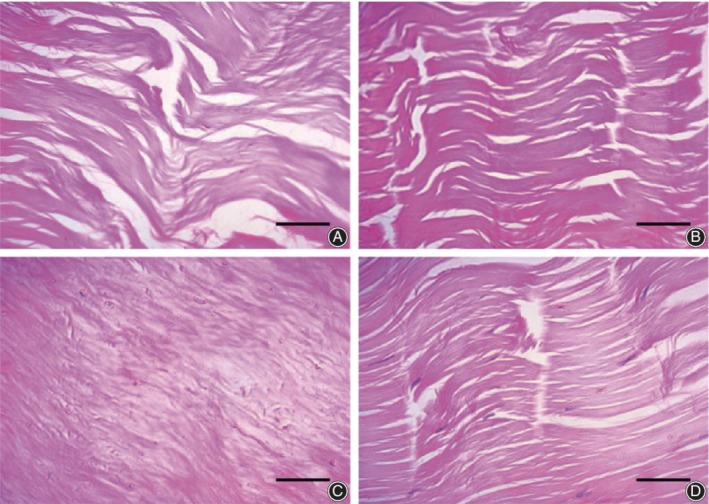
The image showed tendon sections with HE staining. (A) after extraction treatment once, collagen fibers were arranged in parallel, scattered with tendon cells. And loose connective tissue could also be seen. (B) after twice extraction treatments, collagen fibers were still parallel arranged, with loose connective tissue observed, but some of the collagen fibers occasionally interrupted. (C) after three times extraction treatments, collagen bundles were compacted, and the arrangement was disordered. (D) in blank control group, loose connective tissue could be seen between the beams of tendons, and the collagen fibers were arranged in parallel, scattered with tendon cells. ×400, Bar = 40 μm.

**Figure 3:**
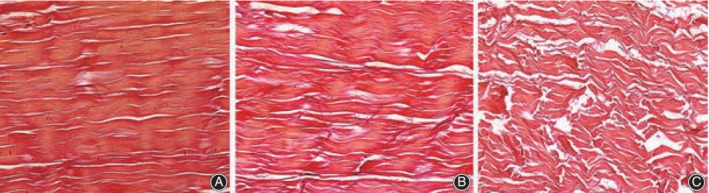
Van Gieson staining of Achilles tendon. (A) treated by one cycle of freezing–thawing, (B) by three cycles, and (C) by 10 cycles (×100).

Optical microscopy is mostly used to visualize collagen fibers and cells. To evaluate the influence of chemical extraction on allogenic tendons, Chen *et al*.^10^ stained tendon specimens with HE and observed the collagen fibers with disordered arrangement and fewer residual cells after repeated chemical extraction treatments (Fig. 2A–D). To evaluate the effect of repeated freeze–thaw cycles on tendons, Chen *et al*.^7^ made histological observations and found that an increase in the disordered arrangement of tendon bundles and collagen fibrils, disrupted cells, and gaps between tendon bundles appeared as the number of freeze–thaw cycles increased (Fig. 3A–C). Quirk *et al*.^17^ stained rat Achilles tendon specimens with HE and safranin orange fast green, and found that in the all frozen group, the tenocytes were smaller and collagen bundles are merged into a uniform appearance. At the enthesis, chondrocytes were best seen with bright purple nuclei and larger, bright cytoplasm in the fresh group. In a morphological study of the anterior cruciate ligament (ACL) and hamstring tendons, Zhu *et al*.^18^ stained tendon specimens with HE, which revealed that, in the near insertion sites of the ACL, the collagen fibers were disordered and loosely arranged.

Using TB stain, Biancalana *et al*.^19^ evaluated the effects of obesity on tendon morphology and found that the metachromasia regions around the cells had a high concentration of proteoglycans. Using the same staining method as used by Biancalana *et al*., Mazon *et al*.^20^ evaluated the effect of different types of resistance exercises on the ECM of tendons. The images showed more intense metachromasia in the distal region of the tendon subjected to compression, also indicating the presence of higher amounts of proteoglycans. Zhu *et al*.^18^ also used TB staining in a morphological study in which the ACL showed stronger positive staining than did hamstring tendons, indicating higher amounts of glycosaminoglycans.

In addition to TB stain, proteoglycans are also evaluated with Alcian blue stain. Mazzocca *et al*.^21^ performed a morphological study investigating tendon pathology in which the positive Alcian blue staining was semi‐quantified with a computed topography system, and the results showed a significant increase in Alcian blue staining, indicating increased tendon degeneration. In general, optical microscopy offers a qualitative evaluation of cells, collagen fibers, and other extracellular matrixes. However, with computed topography, the images can also be semi‐quantified.

#### *Histological Analysis with Scoring Systems*

Many studies have also been conducted with scoring systems to perform semi‐quantitative evaluation. To evaluate the regeneration capacity of rat Achilles tendon allografts, a 0–12 histological scoring system was used to assess seven categories: extracellular matrix organization; cell/matrix ratio; cell distribution; organization of repaired tissue; degenerative changes; vascularization; and inflammation^22^. Using this scoring system, decellularized allografts seeded with tenocytes scored 10 points 24 weeks after the operation, exhibiting better tendon structural recovery than shown by allografts without tenocyte seeding. A 0–21 scoring system was also used in the assessment of tendon ruptures, through which fiber structure, fiber arrangement, rounding of the nuclei, regional variations in cellularity, increased vascularity, decreased collagen stainability, and hyalinization were considered^23^.

## Electron Microscopy

### *Chemical Fixation for the Preparation of SEM and TEM*

Specimen fixation is performed first, then dehydration, embedding into resin, ultramicrotomy, and staining are performed before TEM observations are made, while dehydration and conductive coating are performed before SEM observations are made. Glutaraldehyde, paraformaldehyde, and osmium tetroxide are the most commonly used chemical fixatives used for specimen fixation, and uranyl acetate and lead citrate are usually used as counterstains^19^, ^24^. To remove the outer layer of the tendons to investigate the morphology of human palmaris longus tendons, Nicholls *et al*.^25^ briefly immersed the tendons in 1% v/v acetic acid, which caused outer layers to swell and be separated from the collagen fibers, and then the crimp structures of collagen could be found in SEM images. Similarly, to expose the inner surface, Zhu *et al*.^18^ prepared hamstring tendons and ACL by cryofracture, during which time the specimens were immersed in liquid nitrogen and then fractured with a microsurgical scalpel blade, and observed the smooth surface of the collagen fibers.

For SEM, specimens are commonly sputter coated with gold palladium before imaging^26^. For TEM, ultrathin sectioning, staining or contrasting are applied after embedment^4^, ^7^, ^17^, ^18^. To examine the change in tendon collagen with the administration of glucocorticoids, Taguchi *et al*.^27^ prepared rat Achilles tendon sample slices stained with tannic acid, uranyl acetate, and lead citrate, and the TEM images showed a significant decrease in mean collagen fiber diameter.

### *Ultrastructure Analysis of Tendons* via *SEM and TEM*

#### *Surface and Longitudinal Analysis via SEM*

**Figure 4:**
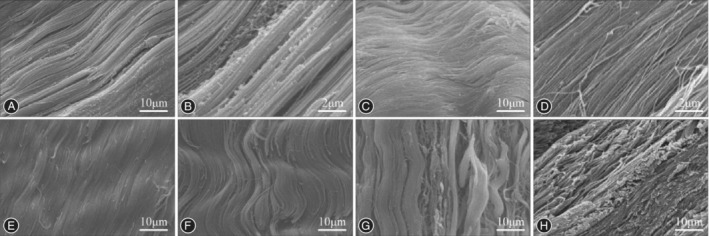
SEM shows the inner surface of hamstring tendons and anterior cruciate ligament (ACL) prepared by cryofracture. The human semitendinosus (A,B) and gracilis tendons (C,D) showed a similar morphology with a smooth surface composed of undulated fibers. (E,F) ACL has a unique type of fiber with helical waveform. (G) Tangled fibers form gaps between sub‐fascicular units. (H) Lamellar layout of ACL in sagittal sections. Original magnification ×1,000 (B and D × 5,000).

When viewed by SEM, the surface and longitudinal section could be evaluated, which in general shows the arrangement of collagen fibers. SEM is often used in the observation and measurement of tendon fiber crimp waves^25^. Zhu *et al*.^18^ observed the inner surface of the ACL and hamstring tendons in SEM and found a smooth surface in hamstring tendons, while a different type of fiber with helical waveform could be seen in ACL (Fig. 4A–E). To evaluate morphological change during aging, Niyomchan *et al*.^16^ evaluated the arrangement of the tendon collagen in human peroneus longus muscle in SEM, and found that the collagen fibers were disoriented and appeared straight without helical coils in an older adult cadaver. Provenzano and Vanderby^28^ evaluated structural change between fetal and skeletally mature rats, while Chen *et al*.^10^ evaluated the influence of chemical extraction on allogenic tendons, with similar results being obtained via SEM and HE staining, showing collagen fibers that had a more disordered arrangement with fewer residual cells after repeated chemical extraction treatments.

SEM is also used in the evaluation of chemical and mechanical treatment to tendons. To investigate the effect of removing glycosaminoglycans with chondroitinase ABC, Franchi *et al*.^29^ prepared Achilles tendons and medial collateral ligaments of the rats, and found collagen fibers interrupted by occasional undulations or crimps of different sizes in SEM images. To evaluate the effect of preconditioning on semitendinosus grafts in ACL reconstruction, Guillard *et al*.^30^ observed SEM images, and based on a semi‐quantitative scoring system (CIP) that accounted for collagen cohesion, integrity, and parallelism, performed an analysis. After preconditioning, the loss of cohesion, integrity, and parallelism of the collagen fibrils was observed in the SEM images with a significant decrease in CIP scores. At higher magnification, Chambers *et al*.^26^ investigated the morphological change after tendon rupture, and the SEM images showed longitudinally distributed discrete kink‐type sites of plastic deformation and frequent loss of D‐banding in the extensor tendons, while the flexor tendons exhibited less damage.

#### *Ultrastructure Analysis of Collagen Fibrils via TEM*

**Figure 5:**
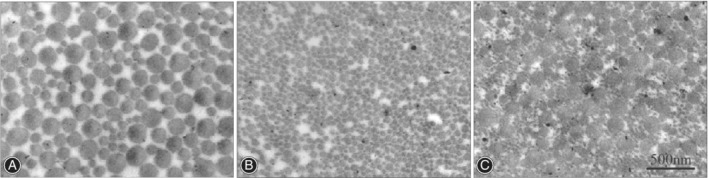
The cross sections of (A) hamstering tendons and (B, C) anterior cruciate ligament (ACL) in TEM images. Bar = 500 nm; original magnification ×40,000.

**Figure 6:**
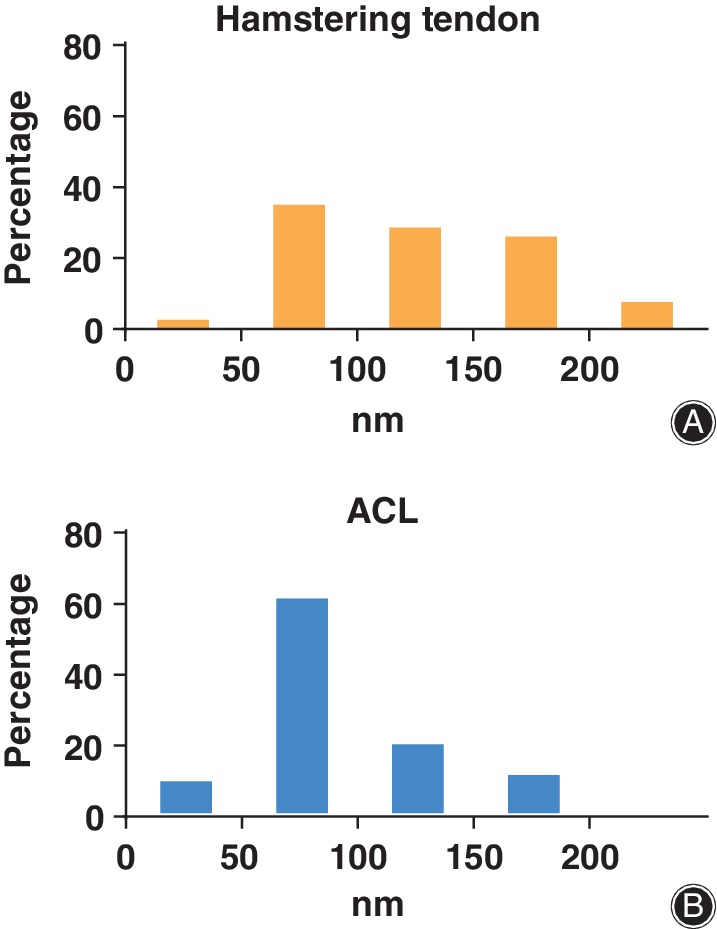
The distribution of fibril diameters in human (A) hamstering tendons and (B) anterior cruciate ligament (ACL).

By TEM, the distribution of collagen fibrils and their diameters are often evaluated in the cross sections of tendons. To evaluate the tendon structure and measure the fibril diameter using TEM images, Andrzejewski *et al*.^31^ massaged the tendons and then classified the fibril diameter as less than 100 nm, 100–200 nm, 200–300 nm, or 300–400 nm and found an increase in the number of collagen fibrils with a diameter less than 100 nm. Zhu *et al*.^18^ made another classification (0–50 nm, 50–100 nm, 100–150 nm, 150–200 nm, and > 200 nm) and found that hamstring tendons had a higher and more asymmetric distribution of fibril diameters, but with lower density, than were observed in the ACLs (Figs 5 and 6). Boesen *et al*.^24^ measured the fibril density, defined as the number of fibrils per square micrometer, and found no difference over time after immobilization.

To investigate the correlation between functional differences among the three bundles of ACL and their microstructures, Suzuki *et al*.^32^ obtained TEM images and calculated the distribution pattern of the fibril diameters and mass average diameter (MAD), which is more representative of tensile strength because it reflects the larger diameter fibrils. The results showed that the MAD of the anteromedial‐lateral bundle was significantly higher than that of the posterolateral bundle. Computer‐assisted tools have been used in the measurement of TEM images. Schmidt *et al*.^33^ trained a machine learning classifier to distinguish collagen fibrils from the background of TEM images and analyzed the distribution of the diameters of collagen fibrils.

TEM is also used in the observation of the longitudinal sections of collagen. Franchi *et al*.^29^ observed isolated collagen fibrils in TEM and found the typical knots or fibrillar crimps and a regular D‐banding of approximately 67 nm. Chen *et al*.^7^ also observed the tendon collagens after repeated freeze–thaw cycles in TEM photographs of the longitudinal section and found that, with the increase in freeze–thaw cycles, the parallel collagen fibrils tended to be compacted and their transverse bands became smaller.

#### *Tendon Evaluation with Both SEM and TEM*

In many studies, TEM and SEM are utilized in combination. For example, using both SEM and TEM, Biancalana *et al*.^19^ investigated the effects of obesity on tendon microstructures. In SEM photomicrographs, undulated collagen fibers and aligned cells were revealed in the compression region, while in the tension region, a network of aligned fibrils formed bundles. Under TEM, the characteristic band pattern in the longitudinal section of collagen and fibrils of different diameters was observed. Under both SEM and TEM, lipid droplets were noted among collagen bundles in the obesity group. Cury *et al*.^14^ evaluated the ultrastructural characteristics of the tendon‐bone junctions in rats, and SEM revealed collagen fibers attached to the bone with higher organization in elderly rats and the distribution of lipid droplets, while TEM showed collagen fibers organized in bands attaching to calcified fibrocartilage, with some tenocytes among them. The high magnification also revealed the striated structure of the collagen fibrils. Franchi *et al*.^34^ investigated the morphology of fibrillar crimp in tendons and ligaments of rats using SEM and TEM images and found that, at the top angle of the crimp region, collagen fibrils twist leftwards changing the plane of running and then sharply bends changing both their direction and plane of coursing.

## Conclusion

Via simple optical microscopy and electron microscopy, the histology and microstructure of tendons could be evaluated. These methods have become the conventional techniques for evaluation of tendon morphology from tissue to collagen fibrils, and are often associated with biomechanical tests. In recent studies, immunohistochemical methods are also used, as well as atomic force microscopy, X‐ray diffraction, and cryo‐TEM, to glean more information on tendon structure. This review presents an overview of the preparation techniques, observation indexes and evaluation methods in optical microscopy and electron microscopy, providing references for tendon morphological studies.

### *Author declaration*

All authors listed above meet the authorship criteria according to the latest guidelines of the International Committee of Medical Journal Editors. All authors agree with the manuscript and have no competing interests.

## Funding

This work was supported by National Key R&D Program of China (No. 2018YFC1106700).
